# The effect of exercise intensity on exercise‐induced hypoalgesia in cancer survivors: A randomized crossover trial

**DOI:** 10.14814/phy2.15047

**Published:** 2021-10-04

**Authors:** Briana K. Clifford, Matthew D. Jones, David Simar, Benjamin K. Barry, David Goldstein

**Affiliations:** ^1^ School of Health Sciences UNSW Sydney Sydney Australia; ^2^ School of Clinical Medicine University of Queensland Brisbane Australia; ^3^ Department of Medical Oncology Prince of Wales Hospital Randwick Australia

**Keywords:** cancer survivor, exercise, intensity, pain, pressure pain threshold

## Abstract

Pain is experienced by people with cancer during treatment and in survivorship. Exercise can have an acute hypoalgesic effect (exercise‐induced hypoalgesia; EIH) in healthy individuals and some chronic pain states. However, EIH, and the moderating effect of exercise intensity, has not been investigated in cancer survivors. This study examined the effect of low‐ and high‐intensity aerobic exercise on EIH in cancer survivors after a single exercise session as well as a brief period of exercise training (2‐weeks, three exercise sessions per week). Participants (*N* = 19) were randomized to low‐ (30%–40% Heart Rate Reserve (HRR) or high‐ (60%–70% HRR) intensity stationary cycling for 15–20 min. Pressure pain thresholds (PPT) were assessed over the *rectus femoris* and *biceps brachii* before and after a single exercise session and again after a short training period at the assigned intensity. Then, following a 6‐week washout period, the intervention was repeated at the other intensity. After the first exercise session, high‐intensity exercise resulted in greater EIH over the *rectus femoris* than low intensity (mean difference ± SE: −0.51 kg/cm^2^ ± 0.15, Cohen's *d* = 0.78, *p* = 0.004). After a 2‐week training period, we found no difference in EIH between intensities (0.01 kg/cm^2^ ± 0.25, *d* = 0.00 *p* = 0.99), with comparable moderate effect sizes for both low‐ and high‐intensity exercise, indicative of EIH. No EIH was observed over the *biceps brachii* of the arm at either low or high intensity. Low‐intensity exercise training may be a feasible option to increase pain thresholds in cancer survivors.

## Graphical abstract

High‐intensity exercise increased pressure pain thresholds more than low‐intensity exercise over the exercising muscle of the leg. However, after a short training period, there was no difference between low‐ and high‐intensity exercise on pain thresholds, with both intensities eliciting a hypoalgesic effect. These results suggest that both low‐ and high‐intensity exercise training may be effective at increasing pain thresholds in cancer survivors.

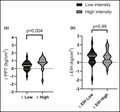

**What is the central question of the study?**

- Is there an effect of exercise intensity on pain thresholds in cancer survivors?

**What is the main finding and its importance?**

- High‐intensity exercise elicits a greater increase in pain thresholds than low intensity after a single bout of exercise. However, after a short 2‐week training period, high‐ and low‐intensity exercise elicit a similar increase in pain thresholds, suggesting that low‐intensity exercise training may be a feasible option to increase pain thresholds in cancer survivors.
- This is important giving the significant barriers to exercise that cancer survivors face, meaning low‐intensity exercise may be a more feasible option.

## 1 INTRODUCTION

Pain is experienced by people with cancer across the cancer continuum. Pain reported in cancer population is highly varied with respect to its cause, its duration, as well as its impact on function, and quality of life. Common cancer treatments such as chemotherapy, radiotherapy, and surgery are all associated with pain syndromes. While most people with cancer experience pain during treatment, some treatments are associated with a more protracted experience of pain well into survivorship. Recent studies suggest that persisting pain may occur in over 50% of cancer survivors across a range of cancer types (Belfer et al., 2013; Bovbjerg et al., 2019; Moye et al., 2014; Mustafa Ali et al., 2017; Prinsloo et al., 2018; Schreier et al., 2018). Additionally, people who have undergone treatment for cancer might have an altered perception of pain or increased pain sensitivity. Unsurprisingly, persistence of pain after treatment is associated with higher self‐perceived disability and lower quality of life (Caro‐Moran et al., 2014). Therefore, strategies to reduce the burden of pain during survivorship are of obvious clinical importance.

Exercise may be one such strategy. Both acute (i.e., a single bout) (Naugle et al., 2012) and chronic exercise (i.e., regular exercise over weeks to months) (Geneen et al., 2017) have been shown to reduce experimentally induced pain in healthy individuals and clinical pain in people with chronic musculoskeletal pain. In the acute setting, a reduction in pain by exercise is known as exercise‐induced hypoalgesia (EIH). EIH typically manifests as a reduction in self‐reported pain during exposure to noxious stimuli and/or as an increase in pain tolerance or pain threshold lasting for <30 min following acute exercise (Naugle et al., 2012; Rice et al., 2019). However, different pain responses have been demonstrated in some chronic pain groups with effects ranging from a blunted EIH response to a hyperalgesic effect of exercise. This indicates that the magnitude of EIH, likely of clinical importance in people with chronic pain, differs within and between chronic pain populations and is often reduced compared to pain free individuals (Rice et al., 2019; Vaegter & Jones, 2020). The intensity of an acute bout of exercise is an important factor in the hypoalgesic effect, with larger EIH observed following higher intensity exercise in healthy individuals (Naugle et al., 2012, 2014). Additionally, the effect of exercise training on the magnitude of EIH has only recently been investigated in healthy populations, where it was shown that EIH increased after a period of exercise training (Hansen et al., 2020).

Despite the prevalence of pain in cancer survivors, the effect of acute and chronic exercise on pain in this cohort has seldom been studied. One of the few studies conducted in cancer survivors investigated the effect of chronic aquatic exercise on pressure pain threshold (PPT) in breast cancer survivors (Cantarero‐Villanueva et al., 2013). They found an increase in PPT (i.e., reduced sensitivity to noxious pressure) after 2 months of hydrotherapy. Similarly, a randomized controlled trial in breast cancer survivors (Fernández‐Lao et al., 2012) reported an increase in PPT after 8 weeks of training consisting of aerobic, strength, and mobility exercises.

No studies to date have investigated the acute effect of exercise or the effect of exercise training on EIH in cancer survivors, nor has the mediating effect of exercise intensity on acute EIH been explored in this population. Therefore, the aim of this study was twofold: (1) to investigate the effect of exercise intensity on EIH after a single bout of exercise, and (2) to investigate the effect of exercise intensity on the magnitude of EIH after a short, 2‐week training period. It was hypothesized that higher intensity exercise would elicit a greater EIH, as evidenced by an increase in pain thresholds, after both acute (single bout) and short‐term (2 weeks) exercise.

## 2 METHODS

This exploratory study was a randomized crossover trial, designed to determine the difference between high‐ and low‐intensity aerobic exercise on experimentally induced pain (pressure pain thresholds) in cancer survivors.

### 2.1 Participants

All procedures were approved by the South Eastern Sydney Local Health District Human Research Ethics Committee (HREC ref 15/170) and conformed to the Declaration of Helsinki, except for registration in a database (2013). Participants were recruited through Prince of Wales Hospital, Department of Oncology. Participants were considered eligible for this study if they: (1) had a diagnosis of non‐metastatic breast cancer, colorectal cancer, prostate cancer, or lymphoma, (2) had completed adjuvant chemotherapy or radiotherapy 3–12 months prior to enrolment, though breast and prostate cancer patients who were on hormonal therapy were eligible to participate, (3) were between the ages of 16 and 80 years, and (4) were currently completing less than 90 min of structured, moderate to vigorous physical activity per week. This last condition ensured that the dose of exercise delivered in the intervention (20 min per session plus warm up and cool down 3× per week) was greater than the participants’ current physical activity levels. Participants were also asked to maintain their usual levels of activity throughout the duration of the study. Potential participants were not required to report pain to be eligible for this study. Participants were excluded if they (1) could not freely give informed consent, (2) were on an experimental drug trial, or (3) were currently using anti‐inflammatory or analgesic medication. Participants who were taking analgesic medications were given the opportunity to participate if they could cease use of the analgesic during the intervention. Eligible participants were identified through the hospital database software, MOSAIQ (Elekta AB), a cancer services information system for electronic medical management. Study information was provided by the patient's treating oncologist. Participants signed a consent to contact if they wanted to be included in the study or if they wanted further information. Once the participants had signed a consent to contact form, they were contacted by a study investigator and, if they were still willing to participate, provided written informed consent. All exercise testing and training took place at the University of New South Wales (Sydney, Australia), Department of Exercise Physiology research testing facility.

### 2.2 Procedures

**FIGURE 1:**
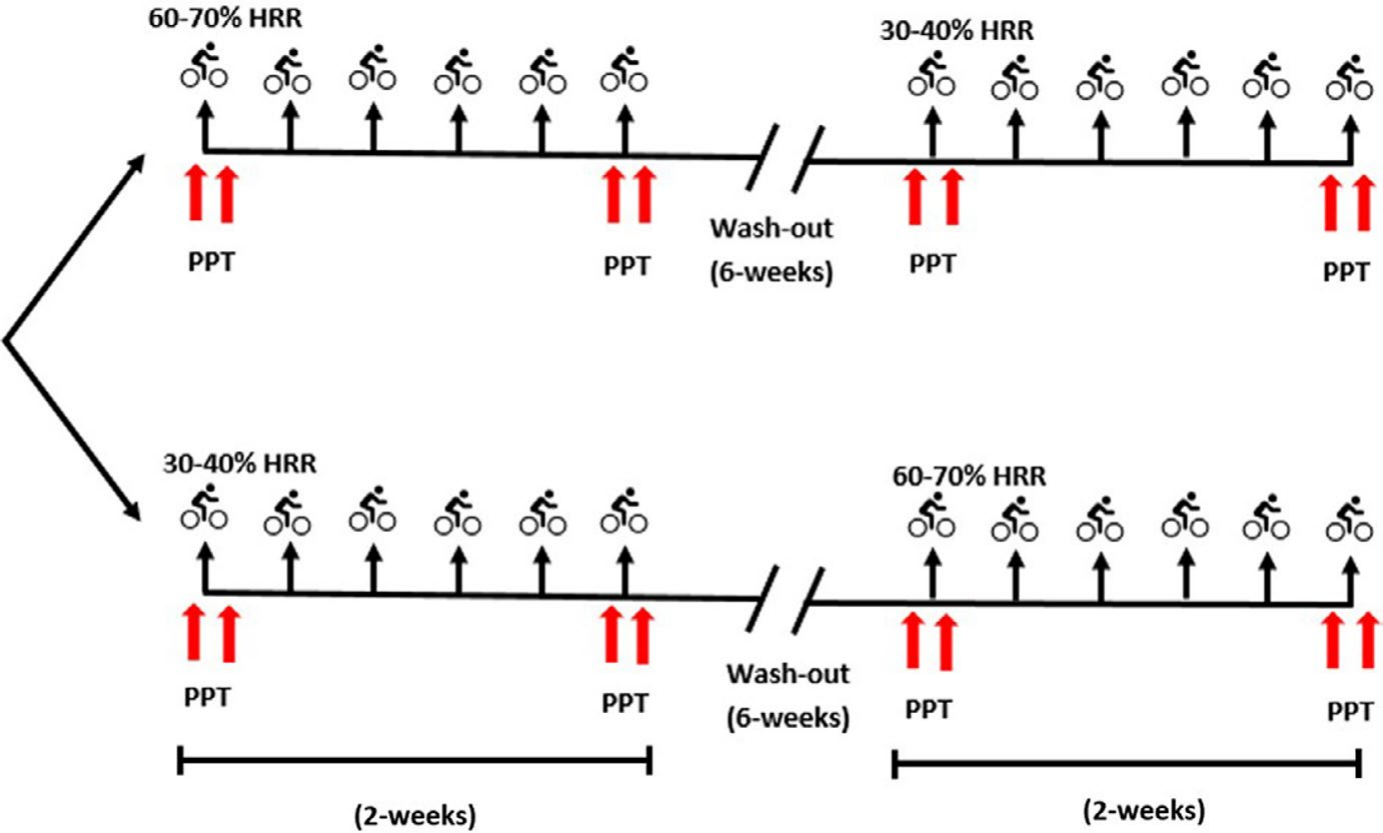
Experimental setup. Participants were randomized to low‐ or high‐intensity exercise and performed 2 weeks of exercise training (stationary bike) at the assigned intensity. PPT (arrows) were assessed immediately before and immediately after the first and last exercise sessions of the 2‐week training period. After the initial 2 weeks of training, participants underwent a 6‐week no exercise washout period and then returned to complete the exercise intervention at the other intensity. HRR, heart rate reserve; PPT, Pressure Pain Threshold

After informed consent was obtained, participants were randomized (using a computer‐generated number sequence from www.randomizer.org) to either the low‐ (30%–40% Heart Rate Reserve (HRR)) or high‐ (60%–70% HRR) intensity exercise group (Figure 1) and underwent baseline aerobic fitness testing on a cycle ergometer (Ergoselect 200, Ergoline GmbH, Lindenstraße 5). Maximal aerobic fitness was predicted using a submaximal aerobic capacity test, the modified YMCA test (Beekley et al., 2004). The modified YMCA test is a graded exercise test performed on a cycle ergometer. Each stage lasts for 3 min and heart rate, blood pressure and rating of perceived exertion are measured at each stage. The test starts at 25 W and workload is increased every 3 min based on the heart rate of the participant. The test is ceased at 80% of the participants age predicted heart rate maximum. Height and weight were measured for each participant at the initial session using a stadiometer (Seca stadiometer 213, Seca) and calibrated scales (Charder Medical), and body mass index (BMI) was calculated. There was 1 week between the baseline testing session and the initial exercise session in the 2 weeks training period. Before each exercise session, participants confirmed that they had not consumed anti‐inflammatory or analgesic medications. The initial exercise session consisted of a 15‐min exercise bout at the prescribed intensity followed by a 5‐min cool down. Heart rate was monitored using a Polar heart rate monitor (Polar Electro Oy) and percentage of HRR was calculated using the equation below:

$${\text{Heart Rate Reserve (HRR) = Intended \textbackslash{}\% of max [Age Predicted HR}}_{\text{max}}{\;\text{(220 - age) - HR}}_{\text{rest}}{\text{] + HR}}_{\text{rest}}$$

At commencement of exercise, the workload was adjusted so that participants reached their assigned intensity within the first 3 min of exercise, after which participants were asked to maintain this intensity for the remainder of the session. Heart rate was continually monitored throughout the training session and recorded every 30 s. Workload was adjusted throughout as necessary to maintain the participant's heart rate within the desired range.

PPTs were measured immediately prior to exercise and immediately after the completion of exercise. In the 2‐week period following the initial exercise bout, participants returned to the lab and completed four exercise sessions. These sessions were increased in length up to 20 min (session 2 = 17 min, sessions 3, 4 & 5 = 20 min), followed by a 5‐min cool down. Participants then returned for the final exercise session, which was matched in duration to the initial session (i.e., 15 min followed by a 5‐min cool down). Participants had at least 1‐day rest between exercise sessions. The duration of the exercise sessions was based on a meta‐analytic review of the hypoalgesic effect of exercise which identified that exercise durations greater than 10 min were more likely to induce a hypoalgesic response (Naugle et al., 2012). PPTs were measured again immediately before and immediately after the final exercise bout. Participants then underwent a washout period of no structured exercise for a minimum of 6 weeks, after which they returned and were crossed over to complete the exercise intervention at the other intensity (Figure 1).

### 2.3 Pressure pain threshold testing

PPTs were measured using algometry, a reliable and valid method for quantifying PPT across a range of healthy and chronic pain populations that is sensitive to the effects of exercise (Chesterton et al., 2007). A pressure algometer (Wagner Force Ten FDX‐25) with a 1‐cm^2^ rubber‐tipped probe was used to apply pressure perpendicularly to the participant's skin. The pressure applied by the algometer was increased at a rate of 1‐kg of force per second until the participant identified that the feeling of pressure had turned to pain. At this point, they gave a verbal command of “stop” and the value displayed on the algometer was recorded as the participant's PPT. The procedure involved a practice trial and was followed by three test trials at each of two sites: the thigh over the *rectus femoris* muscle body and the arm over the mid‐belly of the bicep brachii. The average of the three test trials was determined and recorded as the PPT at each site. Measurements were made from only one arm and leg, determined by: (1) the least affected limb for participants who had lymphoedema (*n* = 3), or (2) the dominant upper limb (*n* = 16). Additionally, it is recognized that delivering specific education regarding EIH can amplify the reported EIH after exercise (Jones et al., 2017; Vaegter et al., 2020). Therefore, no education or information was given about the effect of exercise on pain thresholds until completion of the entire intervention.

### 2.4 SF‐36 Bodily pain subscale

Quality of life was assessed using the RAND 36‐item short form health survey (SF‐36) (Keller et al., 1997). The SF‐36 is a generalized health survey that is used to examine a person's perceived health status across eight health concepts (physical functioning; role limitations because of physical health problems; bodily pain; social functioning; general mental health; role limitations because of emotional problems; vitality and general health perceptions). Possible scores range from 0–100, with a score of 100 representing a very good perceived quality of life. The bodily pain subscale of the SF‐36 was isolated and used in conjunction with PPT to assess clinical pain in the study population at the beginning of the study, and any changes in pain by exercise across the 2‐week training period. Traditionally, the SF‐36 is based on a 4‐week recall period, however, in the present study, a 1‐week recall period version was used, which has been demonstrated to be more sensitive in addressing recent changes to health status (Keller et al., 1997). The SF‐36 was administered at baseline, 1 week before initiation of the 2‐week training period, and at the follow‐up appointment occurring 1 week after the final exercise session.

### 2.5 Sample size calculation

As mentioned previously, few studies have investigated EIH in cancer survivors. Moreover, the available studies in these cohorts have only measured pain before and after a training period and did not capture the acute effect of exercise on pain. Therefore, a meta‐analytic review by Naugle et al. (2012) of EIH in healthy populations was used to identify effect sizes to determine the required sample size for the current study. Naugle et al. (2012) identified two studies (Koltyn et al., 1996; Meeus et al., 2010) that investigated the effect of aerobic exercise on PPTs and the weighted effect size was 0.58. Based on an effect of this size, *n *= 26 was determined as sufficient to capture the acute EIH induced by varying intensities of exercise using the “*t tests* ‐ *Means*: *Difference between two dependent means (matched pairs)”* model [G Power (version: 3.1.9.2) with 80% power and two‐tailed alpha of 0.05].

### 2.6 Statistical analysis

IBM Statistical Package for Social Sciences‐SPSS (version 22) was used for all statistical analyses. Descriptive statistics were calculated, and normality of the data was assessed through histogram analysis of the calculated residuals. A per protocol analysis was completed and a restricted maximum likelihood‐based linear mixed model (LMM) was used to estimate the effect of exercise intensity on pain thresholds, taking into account the within participant correlation of outcome measures. The order (low intensity first vs. high intensity first) and the bout of training (exercise bout 1/before washout vs. exercise bout 2/after washout) effects were adjusted in the model. Results were considered statistically significant if *p* < 0.05. After the difference between intensities was assessed, effect sizes (Cohen's d) were calculated based on the mean difference and standard deviation (SD) at each site, timepoint, and intensity. Effect sizes were interpreted as negligible (<0.2), small (0.2–0.49), moderate (0.5–0.79), or large (>0.8) (Fritz et al., 2012). Paired *t*‐tests were used to assess changes in bodily pain from the SF‐36 bodily pain subscale before and after each 2 week training period, and to assess changes in pre‐exercise pain thresholds after each 2 week training period. Pre‐exercise pain thresholds are identified as those measured immediately before exercise session 1 (Ex1) and immediately before exercise session 6 (Ex6) in each 2 week training period.

## 3 RESULTS

### 3.1 Participant characteristics

**FIGURE 2:**
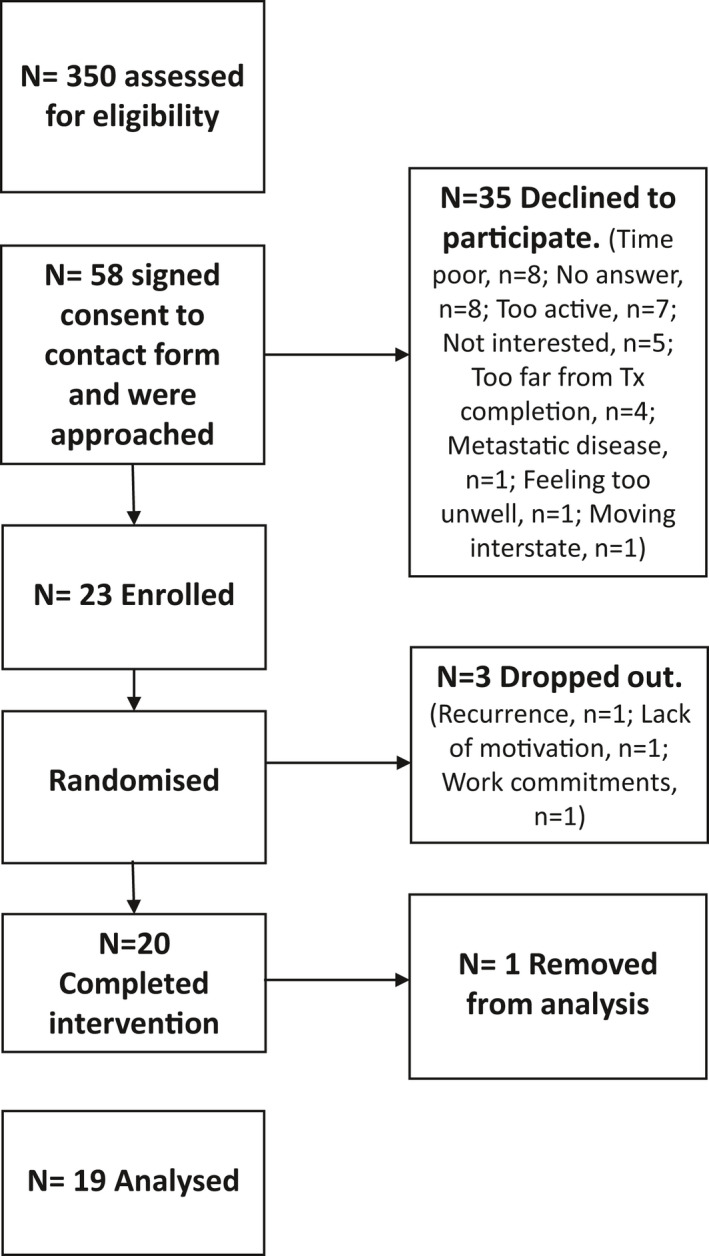
Recruitment diagram detailing flow of participants through the study

Twenty‐three participants were recruited into the study between July 2016 and June 2018 (Figure 2). Three participants dropped out of the study prior to baseline assessment citing a lack of motivation to attend the testing session (*n* = 1), work commitments (*n* = 1), and cancer recurrence (*n* = 1). Twenty participants completed the intervention, however, data for one participant was removed from the analysis as it was significantly outlying data from rest of the group (more than 3 SD from the mean across all timepoints) which significantly skewed the dataset and violated statistical assumptions. The participants, while reporting having been sedentary for approximately 20 years, had a history of elite sport engagement. Previous studies have shown that engagement in elite level sport is associated with reduced pain sensitivity (Tesarz et al., 2012) and may explain the abnormally high pain thresholds. However, we conducted a sensitivity analysis including the outlying data points and this did not significantly change the results (Tables S3 and S4). Participants (*n* = 19) had a mean age of 56.1 years (±9.4), were mostly female breast cancer survivors and were an average of 7.3 month from completion of adjuvant therapy (Table 1). The average intensity achieved during the high‐intensity training sessions was 65.2 ± 7.2% of heart rate reserve (HRR; target 60%–70% HRR) and during low‐intensity training was 31.4 ± 9.4% HRR (target 30%–40% HRR). On average, participants competed 97.5% of exercise sessions. No adverse events were reported during the study.

**TABLE 1:** Participant characteristics

	Baseline (*n* = 19)	Baseline 2 (*n* = 19)	Mean diff	*p*‐value
Age (years)	56.1 ± 9.4	—	—	—
Sex	86% Female	—	—	—
Diagnosis	60% Breast cancer; 27% Colorectal cancer; 13% Prostate cancer	—	—	—
Time since treatment (months)	7.3 ± 4.5	—	—	—
Height (cm)	167.1 ± 5.4	—	—	—
Weight (kg)	81.5 ± 23.4	83.8 ± 23.1	2.30	0.17
BMI (kg/m^2^)	29.4 ± 8.1	30.1 ± 8.1	0.6	0.16

### 3.2 Effect of exercise intensity on pain thresholds before and after short‐term exercise training over the rectus femoris

**FIGURE 3:**
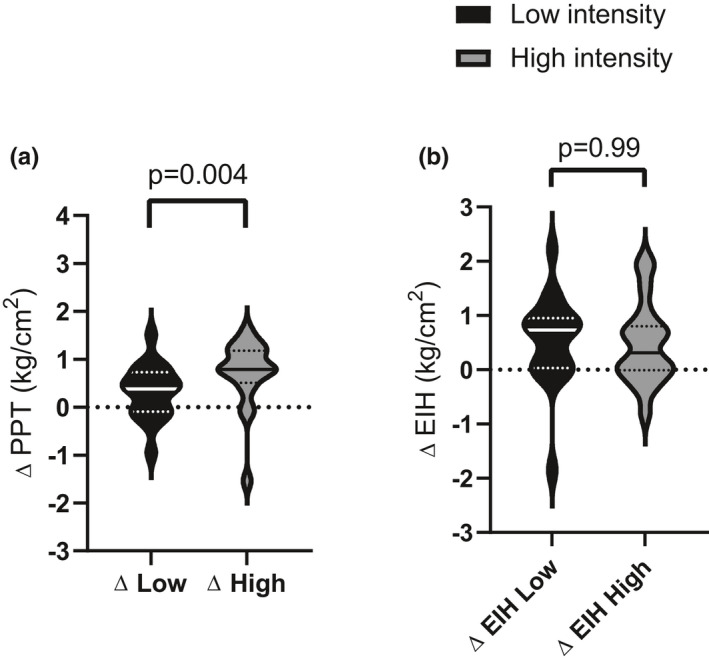
Effect of acute exercise and short‐term exercise training on pain thresholds over the rectus femoris. (a) Acute effect of exercise on PPT: Change in PPT over the rectus femoris, before and after exercise session 1 for each intensity. (b) Effect of short‐term training on the magnitude of EIH over the rectus femoris: EIH represents the change in pre‐post PPT before and after the exercise intervention. The unbroken center line indicates the median, perforated lines indicate quartiles and the extremities represent the range

After a single bout of exercise, we found a significant difference between exercise intensities on PPT over the *rectus femoris* (mean difference between intensities [post exercise ‐ pre exercise] ± SE: −0.51 kg/cm^2^ ± 0.15, *p* = 0.004) in favor of high intensity (Figure 3, panel a). High‐intensity exercise elicited a moderate effect size over the *rectus femoris* while low intensity elicited a small effect size over the *rectus femoris* (Table 2). We found no effect of the order of the intervention or the bout of training (e.g., high or low intensity first, before or after the washout period [Table S1]).

**TABLE 2:** Mean (standard deviation) pre‐ and post‐exercise pressure pain thresholds before and after a short training period

Site	Intensity	PPT Pre Ex1 kg/m^2^	PPT Post Ex1 kg/m^2^	*∆*	Cohen's *d* Effect size	PPT Pre Ex6 kg/m^2^	PPT Post Ex6 kg/m^2^	*∆*	Cohen's *d* Effect size
Rectus Femoris	Low	5.32 (1.82)	5.64 (0.52)	0.52 (0.55)	0.23	5.12 (1.64)	5.98 (1.74)	0.86 (0.59)	0.51
	High	5.09 (1.29)	5.85 (1.32)	0.76 (0.68)	0.58	5.28 (1.74)	6.35 (1.98)	1.26 (0.7)	0.57
Biceps Brachii	Low	2.27 (0.80)	2.41 (0.73)	0.14 (0.26)	0.18	2.29 (0.66)	2.50 (0.78)	0.21 (0.32)	0.29
	High	2.39 (0.72)	2.51 (0.81)	0.12 (0.40)	0.15	2.43 (0.95)	2.76 (0.93)	0.33 (0.50)	0.35

We then assessed the effect of a short training period on baseline PPT and found no difference in pre‐exercise pain thresholds between low intensity (mean *Δ* ± SD: 0.20 kg/m^2^ ± 1.25 kg/m^2^, *p* = 0.50) and high intensity (mean *Δ* ± SD: −0.23 kg/m^2^ ± 1.22kg/m^2^, *p* = 0.44) over the *rectus femoris*.

Finally, we assessed the effect of a short training period (2 weeks) at high and low intensity on the acute EIH response, that is, the effect of 2 weeks exercise training on experimental pain responses to a single exercise bout of exercise. After a short training period there was no difference between high‐ and low‐intensity exercise on acute EIH over the *rectus femoris* (mean difference between intensities ± SE: 0.01 kg/cm^2^ ± 0.25, *p* = 0.99, Figure 3, panel b). Both low‐ and high‐intensity exercise elicited a moderate effect on EIH over the *rectus femoris* (Table 2).

### 3.3 Effect of exercise intensity on pain thresholds before and after short‐term exercise training over the biceps brachii

**FIGURE 4:**
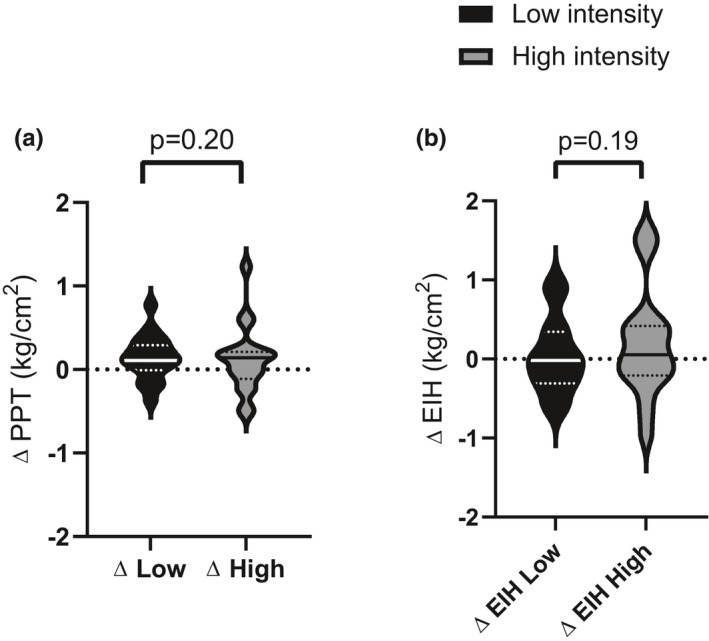
Effect of acute exercise and short‐term exercise training on pain thresholds over the biceps brachii. (a) Acute effect of exercise on PPT: Change in PPT over the biceps brachii, before and after exercise session 1 for each intensity. (b**)** Effect of short‐term training on the magnitude of EIH over the biceps brachii: EIH represents the change in pre‐post PPT before and after the exercise intervention. The unbroken center line indicates the median, perforated lines indicate quartiles and the extremities represent the range

There was no significant effect of intensity, order, or bout of training on PPT over the *biceps brachii* after a single bout of exercise (Figure 4, Table S1).

There was also no difference between the effect of high‐ and low‐intensity exercise training on acute EIH over the *biceps brachii* (mean difference between intensities ± SE: −0.20 kg/cm^2^ ± 0.14, *p* = 0.18, Table S2). Both high‐ and low‐intensity exercise elicited a small effect on EIH over the *biceps brachii* after training demonstrating no systemic effect of exercise on EIH (Figure 4, Table 2).

High intensity elicited a moderate effect on PPT over the *rectus femoris*, the primary exercising muscle, after both the first and last exercise session in a short 2 week training period (Table 2). Low‐intensity exercise elicited only a small effect on PPT over the *rectus femoris* after the first exercise session, but a moderate effect after the final exercise session in a short 2‐week training period (Table 2). Both low‐ and high‐intensity exercise elicited a negligible effect on PPT over the *biceps brachii* (non‐exercising muscle) after a single bout of exercise (Ex1) and a small effect after a short training period (Ex6) (Table 2).

### 3.4 SF‐36‐bodily pain subscale

No change in bodily pain was reported after high‐intensity exercise (mean *Δ* ± SD: −3.33 ± 12.49, *p *= 0.32) or low‐intensity exercise (mean *Δ* ± SD: −0.33 ± 14.6, *p *= 0.93) after 2 weeks of training.

## 4 DISCUSSION

This study demonstrated that high‐intensity aerobic exercise has a greater effect on pain thresholds than low intensity after a single bout of exercise in cancer survivors. However, after a short training period, low‐intensity exercise elicited a similar hypoalgesic effect as high intensity over the working muscle. Our results provide novel knowledge to the limited literature on exercise‐induced pain moderation in cancer survivors in several ways. First, it is the only study investigating the acute effect of exercise on pain perception in cancer survivors and the mediating effect of exercise intensity on the magnitude of EIH. Second, this is the first study to investigate the effect of a short training period on the magnitude of the acute change in pain thresholds after exercise in cancer survivors. Additionally, we investigated the effect of different intensities of exercise on this short‐term training response, which is novel.

We hypothesized that EIH would be higher in response to high‐intensity compared to low‐intensity exercise. In partial agreement with this hypothesis, our results showed a significant effect of intensity on reducing pain sensitivity over the *rectus femoris* after an acute bout of exercise. That is, sensitivity to mechanically induced pain decreased over the leg after high‐intensity exercise, whereas sensitivity was not reduced after low‐intensity exercise. An intensity‐dependent effect of acute exercise on EIH has previously been reported in healthy populations, with moderate‐high‐intensity aerobic exercise (60%–75% maximal oxygen uptake) of longer durations (> 10 min up to 30 min) more likely to elicit EIH (Hoffman et al., 2004; Koltyn, 2002; Naugle et al., 2012). This could explain why no EIH was observed following the initial low‐intensity exercise session in the present study.

While the acute effect of exercise on EIH has been well demonstrated in healthy populations (Naugle et al., 2012), few studies have focused on the effect of chronic exercise on the EIH response. One such study investigated the effect of exercise training on acute exercise‐induced pain flares in people with hip and knee pain (Sandal et al., 2016). This study found that exercise training reduced the magnitude of exercise‐induced pain flares across the course of the 8 week training period. Although this observation was made in the context of clinical pain rather than experimentally induced pain, the results are in line with our current findings. Here, we demonstrated that the effect of exercise on EIH increased after a short training period in both low‐ and high‐intensity conditions for EIH.

Interestingly, our results showed that after 2 weeks of training, the difference between exercise intensities on EIH was no longer observable. Indeed, after a short period of training, both high‐ and low‐intensity exercise elicited a similar change in PPT after a single bout of exercise. This novel finding suggests that, while acute EIH may be initially greater with high‐intensity exercise, short‐term exercise at low intensity can ultimately elicit a similar hypoalgesic response after only a few weeks. This could have significant implications for cancer survivors who are often unable to engage in higher intensity exercise due to barriers such as pain and fatigue (Clifford et al., 2018).

While an intensity‐dependent effect on EIH was observed over the working muscle of the leg in this study, there was no effect of exercise on EIH over the arm (non‐exercised limb) at either high‐ or low‐intensity exercise. In healthy individuals, EIH can be experienced in non‐exercised limbs, albeit usually to a smaller extent (Jones et al., 2017). In agreement with previous research (Micalos & Arendt‐Nielsen, 2016; Vaegter et al., 2014), in the present study where stationary cycling was utilized, exercise had a larger effect on the exercising limb compared to the non‐exercised limb, regardless of the intensity of exercise, with only small EIH observed in the non‐exercised limb. This minimal “systemic effect” of exercise on pain may be a result of insufficient stimulus with respect to the duration and/or intensity of exercise. This may be an important consideration for cancer‐specific cohorts. For example, our cohort was predominantly breast cancer survivors who are more likely to experience ongoing upper limb and chest wall pain (Sagen et al., 2014). In this circumstance, exercise prescription may need to be tailored to target the regions most affected by pain. We also saw no change in self‐reported bodily pain as measured by the SF‐36 after either low‐ or high‐intensity exercise. This could be explained by the limited duration of each exercise intervention as previous research has shown that longer duration interventions (>4 weeks) exhibit a greater reduction in bodily pain (Geneen et al., 2017).

A number of potential mechanisms have been suggested for the effect of exercise on pain thresholds (Jones, Taylor, et al., 2017). During acute exercise, the release of endogenous substances contribute to the hypoalgesic response through peripheral and central mechanisms (Koltyn et al., 2014). Previous research in animal models has demonstrated that exercise increases endogenous opioid concentration suggesting central control of pain modulation affected by exercise training (Stagg et al., 2011). Furthermore, our team has demonstrated that the occlusion of blood flow to exercised limbs attenuates EIH, suggesting that neurotransmitters released during exercise may also act peripherally to influence EIH (Jones, Taylor, et al., 2017). The release of these endogenous neurotransmitters in response to exercise may be influenced by the intensity and duration of exercise. Thus, it could be speculated that a combination of central and peripheral mechanisms contribute to the effect demonstrated in this study. However, further research is required to understand the interaction of these mechanisms.

The cohort in this study reported varying levels of clinical pain at baseline and there was no change in clinical pain reported across the 2‐week training intervention at either low‐ or high‐intensity. This was supported by the lack of change in baseline pain thresholds (Pre Ex1 vs. Pre Ex6) after the 2‐week training period at either high or low intensity. While the impact of exercise at varying intensities on clinical pain is an important outcome, it should be noted that 2 weeks of exercise may not be sufficient to reduce clinical pain in this population. Indeed, previous studies showing reduced pain in cancer survivor populations with exercise training at least 8 weeks long (Cantarero‐Villanueva et al., 2013; Fernández‐Lao et al., 2012). Additionally, participants in the present study were previously sedentary. Research has shown that initiation of exercise may be associated with a transient increase in muscle pain and discomfort which resolves with adaptation to exercise (Geneen et al., 2017). As such, the effect of this short training period on clinical pain may not predict the effect of a longer intervention on clinical pain at low and high intensity, and further investigation of these adaptations is warranted.

### 4.1 Limitations

Our novel findings should be considered in the context of a limited sample size as difficulties in recruitment resulted in a smaller than proposed sample size. An underpowered study can increase the likelihood of type I and type II errors occurring (Fletcher, 2008). However, moderate effect sizes were observed for some of the effects, suggesting that it is unlikely that type II error occurred. Nonetheless, a larger sample size would increase confidence in the findings of this study. The lack of a non‐exercising control group limited our ability to assess the effect of each individual exercise intervention on pain sensitivity in this cohort and should be assessed in future randomized controlled trials. Additionally, in this study potential mechanisms of EIH, including intensity‐dependent differences, could not be established.

### 4.2 Future directions

Future research should investigate the effect of exercise on several candidate biologic systems including circulating endogenous factors such as opioids and other nociceptive mediators, to better understand the mechanisms underlying the analgesic effect of exercise (Marchand et al., 2005).

Understanding the impact of exercise and specific education (Vaegter et al., 2020) on pain sensitivity could maximize the benefits of exercise for pain management in this group and should be a focus of future research. Recent evidence suggests that specific education about the hypoalgesic effect of exercise can impact EIH (Jones, Valenzuela, et al., 2017; Vaegter et al., 2020) and impact pain intensity in cancer survivors (Pas et al., 2020). This is particularly relevant for cancer survivors who may be avoidant of exercise due to fear of worsened pain symptoms. A combination of low‐intensity exercise and pain education may be one way to maximize the beneficial impacts of exercise on pain in this population.

In addition to pain education, the use of resistance training to modulate EIH was not investigated in this study. Resistance exercise is considered safe and effective for cancer survivors and has been shown to increase pain thresholds in healthy and other chronic disease populations (Li et al., 2016). Future research is needed to investigate the effects of resistance exercise of varying intensities on pain thresholds in cancer survivors.

## 5 CONCLUSION

Our data identified anatomical site‐specific and intensity‐dependent effects of exercise on experimental pain sensitivity in cancer survivors. Additionally, we demonstrated the absence of intensity‐specific effect on pain sensitivity after a short training period. Our findings suggest that even low‐intensity aerobic exercise may have hypoalgesic benefits for cancer survivors who are less able or willing to exercise at high intensities. These results are encouraging, since many survivors report treatment‐related side effects, such as fatigue and pain, as barriers to exercise for which low‐intensity exercise may be a more viable option. However, replication of these findings should be a priority in future research to confirm the reliability of EIH in cancer survivor cohorts. Following this, further investigation into the impact of different intensities of exercise on clinical pain and the combined effect of exercise and specific education about EIH could help to maximize the effects seen in this study.

## CONFLICTS OF INTEREST

The authors of this article have no relevant financial or non‐financial conflict of interest to declare.

## AUTHOR CONTRIBUTION

All authors contributed to the study conception and design. Material preparation, data collection, and analysis were performed by Briana K. Clifford, David Simar, Benjamin K. Barry, and David Goldstein. The first draft of the manuscript was written by Briana K. Clifford and all authors commented on previous versions of the manuscript. All authors read and approved the final manuscript.

## ETHICS

This study was approved by the South Eastern Sydney Local Health District, Human Research Ethics Committee (HREC reference: 17‐079).

## CONSENT TO PARTICIPATE

Informed consent was obtained from all individual participants included in the study.

## Supporting information



Supplementary Material

## Data Availability

The data that support the findings of this study are available on request from the corresponding author. The data are not publicly available due to privacy or ethical restrictions.
