# Supplementary material for: The effect of exercise intensity on exercise‐induced hypoalgesia in cancer survivors: A randomized crossover trial
Source: Physiol Rep. 2021 Oct 4;9(19):e15047. doi: 10.14814/phy2.15047 (PMC8488554; doi:10.14814/phy2.15047)
Supplement: Supplementary file 1 — Supplementary Material [file PHY2-9-e15047-s001.docx]

**Supplementary Table 1 – Acute exercise-induced hypoalgesia over the leg and arm.**

| Intensity | Fixed Effect | Mean difference (kg/cm^2^) | Std. Error | 95% Confidence interval | | P-value | Cohen’s *d* |
| --- | --- | --- | --- | --- | --- | --- | --- |
|  |  |  |  | **Lower bound** | **Upper bound** |  |  |
| Rectus Femoris | **Intensity** | **-0.51** | **0.15** | **-0.826** | **-0.190** | **<0.01*** | **0.78** |
|  | Order | -0.27 | 0.16 | -0.615 | 0.080 | 0.12 | 0.39 |
|  | Bout | -0.31 | 0.15 | -0.628 | 0.008 | 0.06 | 0.47 |
| Biceps Brachii | **Intensity** | **0.00** | **0.09** | **-0.194** | **0.200** | **0.20** | **0.00** |
|  | Order | 0.07 | 0.14 | -0.229 | 0.379 | 0.61 | 0.11 |
|  | Bout | 0.00 | 0.09 | -0.192 | 0.202 | 0.96 | 0.00 |

*Linear mixed model analysis: Estimate of fixed effects: Acute exercise-induced hypoalgesia over rectus femoris and the biceps brachii. Dependent variable: Difference between intensities, * statistical significance.*

**Supplementary Table 2 – Effect of short training period on acute exercise-induced hypoalgesia over the leg and arm.**

| Intensity | Fixed Effect | Mean difference (kg/cm^2^) | Std. Error | 95% Confidence interval | | P-value | Cohen’s *d* |
| --- | --- | --- | --- | --- | --- | --- | --- |
|  |  |  |  | **Lower bound** | **Upper bound** |  |  |
| Rectus Femoris | **Intensity** | **0.01** | **0.25** | **-0.531** | **0.540** | **0.99** | **0.01** |
|  | Order | 0.35 | 0.28 | -0.242 | 0.946 | 0.23 | 0.20 |
|  | Bout | 0.30 | 0.25 | -0.232 | 0.838 | 0.25 | 0.27 |
| Biceps Brachii | **Intensity** | **-0.20** | **0.14** | **-0.502** | **0.103** | **0.18** | **0.33** |
|  | Order | -0.29 | 0.23 | -0.769 | 0.181 | 0.20 | 0.29 |
|  | Bout | 0.04 | 0.14 | -0.259 | 0.346 | 0.77 | 0.07 |

*Linear mixed model analysis: Estimate of fixed effects: - Effect of 2-weeks of exercise training on acute exercise induced hypoalgesia over rectus femoris and the biceps brachii. Dependent variable: Difference between intensities.*

**Supplementary Table 3 – Acute exercise-induced hypoalgesia over the leg and arm including outlier.**

| Intensity | Fixed Effect | Mean difference (kg/cm^2^) | Std. Error | 95% Confidence interval | | P-value | Cohen’s *d* |
| --- | --- | --- | --- | --- | --- | --- | --- |
|  |  |  |  | **Lower bound** | **Upper bound** |  |  |
| Rectus Femoris | **Intensity** | **-0.48** | **0.14** | **-0.771** | **-0.179** | **<0.01*** | **0.76** |
|  | Order | -0.64 | 0.38 | -1.430 | 0.159 | 0.11 | 0.37 |
|  | Bout | -0.32 | 0.14 | -0.620 | -0.027 | 0.03* | 0.51 |
| Biceps Brachii | **Intensity** | **0.10** | **0.11** | **-0.122** | **0.319** | **0.36** | **0.20** |
|  | Order | -0.02 | 0.15 | -0.331 | 0.292 | 0.90 | 0.03 |
|  | Bout | -0.08 | 0.11 | -0.301 | 0.146 | 0.48 | 0.16 |

*Linear mixed model analysis: Estimate of fixed effects: Acute exercise-induced hypoalgesia over rectus femoris and the biceps brachii. Dependent variable: Difference between intensities, * statistical significance.*

**Supplementary Table 4 – Effect of short training period on acute exercise-induced hypoalgesia over the leg and arm including outlier.**

| Intensity | Fixed Effect | Mean difference (kg/cm^2^) | Std. Error | 95% Confidence interval | | P-value | Cohen’s *d* |
| --- | --- | --- | --- | --- | --- | --- | --- |
|  |  |  |  | **Lower bound** | **Upper bound** |  |  |
| Rectus Femoris | **Intensity** | **-0.30** | **0.29** | **-0.538** | **0.657** | **0.84** | **0.23** |
|  | Order | 0.43 | 0.27 | -0.138 | 0.998 | 0.23 | 0.36 |
|  | Bout | 0.58 | 0.33 | -0.109 | 1.263 | 0.09* | 0.39 |
| Biceps Brachii | **Intensity** | **-0.22** | **0.14** | **-0.509** | **0.060** | **0.11** | **0.35** |
|  | Order | -0.21 | 0.22 | -0.673 | 0.257 | 0.36 | 0.21 |
|  | Bout | 0.06 | 0.14 | -0.215 | 0.353 | 0.62 | 0.10 |

*Linear mixed model analysis: Estimate of fixed effects: - Effect of 2-weeks of exercise training on acute exercise induced hypoalgesia over rectus femoris and the biceps brachii. Dependent variable: Difference between intensities.*

**Supplementary Figure 1 –Pressure pain thresholds before and after training over the leg and arm, including outlier.**


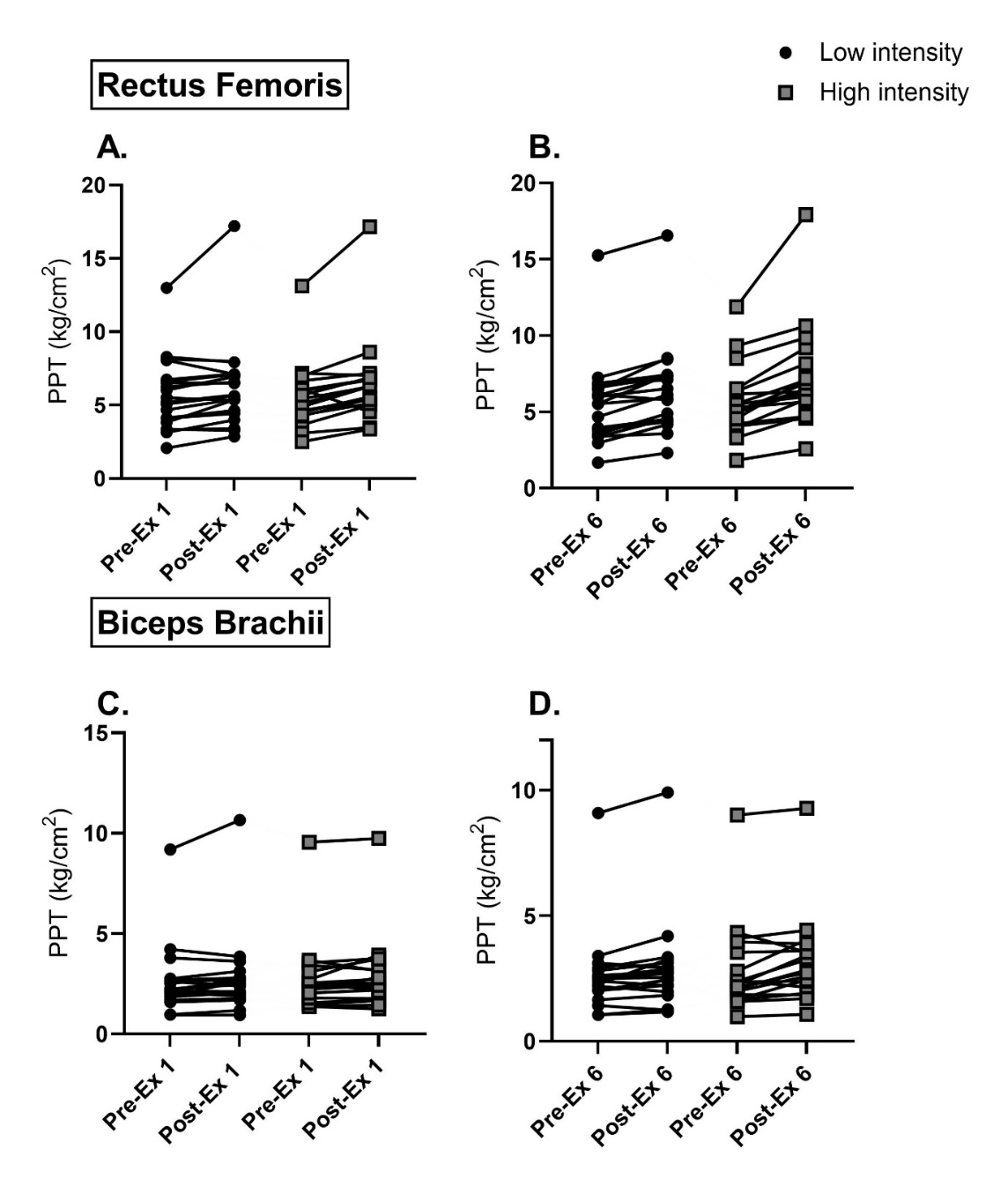


***A.*** *Individual data points for PPT before and after exercise session 1 for each intensity over the leg.* ***B.*** *Individual data points for PPT before and after exercise session 6 (post 2-weeks of training) for each intensity over the leg.* ***C.*** *Individual data points for PPT before and after exercise session 1 for each intensity over the arm.* ***D.*** *Individual data points for PPT before and after exercise session 6 (post 2-weeks of training) for each intensity over the arm.*
